# Association of body mass index with survival in U.S. cancer survivors: a cross-sectional study of NHANES 1999–2018

**DOI:** 10.3389/fonc.2023.1180442

**Published:** 2023-05-12

**Authors:** Yi Yang, Dan Chen, Dingfu Zhong, Zongbi Yi

**Affiliations:** ^1^ Department of Gastroenterology, Jinhua People’s Hospital, Jinhua, Zhejiang, China; ^2^ Hubei Key Laboratory of Tumor Biological Behaviors, Department of Radiation and Medical Oncology, Hubei Cancer Clinical Study Center, Zhongnan Hospital of Wuhan University, Wuhan, China

**Keywords:** body mass index (BMI), all-cause mortality, obesity, cancer survivors, National Health and Nutrition Examination Survey (NHANES)

## Abstract

**Background:**

Understanding the association between relative mortality with body mass index (BMI) may aid clinicians in making suitable clinical decisions. Our study evaluated the impact of BMI on mortality among cancer survivors.

**Methods:**

We used data from the US National Health and Nutrition Examination Surveys (NHANES) spanning from 1999 to 2018. Relevant mortality data were retrieved up until December 31, 2019. Adjusted Cox models were employed to examine the association of BMI with the risks for total and cause-specific mortality.

**Results:**

Among 4135 cancer survivors, 1486 (35.9%) were obese (21.0% class 1 obesity [BMI 30-< 35 kg/m^2^], 9.2% class 2 obesity [BMI 35 -< 40 kg/m^2^], 5.7% class 3 obesity [BMI ≥ 40 kg/m^2^]), 1475(35.7%) were overweight (BMI 25-< 30 kg/m^2^). During an average follow-up of 8.9 years (35895 person-years), a total of 1361 deaths were reported (cancer 392; 356 cardiovascular disease [CVD]; 613, non-cancer, non-CVD). In multivariable models, underweight participants (BMI < 18.5 kg/m^2^) were associated with significantly higher risks of cancer-specific (HR, 3.31; 95% CI, 1.37-8.03, *P*=0.01) and CVD cause (HR, 3.18; 95% CI, 1.44-7.02, *P* < 0.001) mortality compared to individuals with normal weight. Being overweight was associated with significantly lower risks of non-cancer, non-CVD cause mortality (HR, 0.66; 95% CI, 0.51-0.87, *P* < 0.001). Class 1 obesity was associated with significantly reduced risks of all-cause (HR, 0.78; 95% CI, 0.61-0.99, *P* = 0.04), and non-cancer, non-CVD cause (HR, 0.60; 95% CI, 0.42-0.86, *P* = 0.01) mortality. A higher risk of CVD-related mortality (HR, 2.35; 95% CI, 1.07-5.18, *P* = 0.03) was observed in class 3 obesity cases. Lower risks of all-cause mortality were detected in men (overweight, HR, 0.76; 95% CI, 0.59-0.99, *P*=0.04; class 1 obesity, HR, 0.69; 95% CI, 0.49-0.98, *P* = 0.04) but not in woman, in never-smokers (class 1 obesity, HR, 0.61; 95% CI, 0.41-0.90, *P*=0.01) and former smokers (overweight, HR, 0.77; 95% CI, 0.60-0.98, *P*=0.04) but not in current smokers; in obesity-related cancer (class 2 obesity, HR, 0.49; 95% CI, 0.27-0.89, *P*=0.01) but not in non-obesity-related cancers.

**Conclusions:**

In the United States, cancer survivors with overweight or moderate obesity (class 1 or class 2 obesity) demonstrated a lower risk of all-cause and noncancer, non-CVD cause mortality.

## Introduction

A 33% increase in the global incidence of cancer was reported between 2005 and 2015 (1). In the United States, the population of cancer survivors is approximately 15.5 million, and this number is projected to reach up to 22.1 million by 2030 (2). The growing number of cancer survivors has underscored the urgent need to establish standards for survivorship care to improve their long-term health outcomes. Weight management plays a crucial role in cancer survivors, as several clinical guidelines suggest that a higher body mass index (BMI) is associated with poorer survival among this population. Consequently, they are advised to achieve or maintain a normal body weight (3–5). Increased BMI has been shown to be significantly associated with a heightened risk of morbidity and mortality in conditions such as cardiovascular disease (CVD), specific types of cancer, and metabolic diseases (6–8). However, numerous studies have proposed that elevated BMI may lead to an “obesity paradox,” which confers a survival advantage among patients with chronic diseases due to increased BMI (9–11).

A survival benefit has been reported in overweight and moderately obese cancer survivors which supports the obesity paradox associated with cancer (12–15). This phenomenon is less recognized in cancer and presents disputable explanations (16–18). Inconsistent results in previous studies could be attributed to methodological choices (e.g., whether the study accounts for collider bias and confounding due to smoking) and biologically plausible clinical explanations, as well as true causal association. Therefore, this study aimed to exam the association between BMI and survival among cancer survivors in the United States.

## Methods

### Study design and population

In this study, data were procured from 10 survey cycles of NHANES spanning from 1999 to 2018 (19). The National Center for Health Statistics (NCHS) and Center for Disease Control and Prevention (CDC) have conducted a 2-year cycle survey since 1999 to assess the health and nutritional status of the civilian US population. The NHANES protocols were approved by the NCHS ethics review board, and informed consent was obtained from all study participants. Subject characteristics were collected during the body measurement examinations. BMI was calculated as weight (kg)/(height [(m])^2^). According to the standard WHO criteria, BMI was categorized into four groups (underweight BMI < 18.5 kg/m^2^, the normal weight 18.5 to < 25 kg/m^2^, overweight 25 to <30 kg/m^2^, obesity ≥ 30 kg/m^2^) (20). Obesity was further classified into three categories: class 1 obesity (BMI 30 to < 35 kg/m^2^), class 2 obesity (BMI 35 to < 40 kg/m^2^), and class 3 obesity (BMI ≥ 40.0 kg/m^2^) (21).

### Diagnosis of cancer

Date on self-reported history of cancer were obtained from “medical conditions” section of NHANES which contain information on health conditions. Cancer survivors were identified based on the response to the question: “Have you ever been told by a doctor or other health professional that you had cancer or malignancy of any kind?”. The age at the time of the first cancer diagnosis was collected by asking, “How old were you when cancer was first diagnosed?”. The years since cancer diagnosis was calculated as the difference between participants’ current age and their age at first cancer diagnosis, and they were subsequently categorized into (< 2 years, 2 to < 5 years, 5 to < 10 years, and ≥ 10 years) (22). Cancer types were confirmed by asking, “What kind of cancer was it?” and were classified into obesity-related (cancers of the colon, breast, esophagus, rectum, pancreas, gallbladder, stomach, liver, kidney, blood, uterus, and ovary) and non-obesity-related (lung, prostate, cervix, uterus, melanoma, skin, lymphoma/Hodgkin’s disease, and thyroid) cancers (23).

### Ascertainment of mortality

Mortality data were collected from National Death Index up to December 31, 2019. The cause of death was recorded following ICD-10 (International Statistical Classification of Diseases, 10th revision) codes. The primary outcomes included cancer mortality (ICD-10 codes C00-C97) and CVD-cause mortality, encompassing heart disease (ICD-10 codes I00-I09, I11, I13, I20-I51) and cerebrovascular diseases (ICD-10 codes I60-I69) The mortality follow-up time was calculated from the date when the body measurements were taken to the date of patient’s death or December 31, 2019 (24). To reduce the probability of reverse causality, we excluded the participants who died within the first 24 months of follow-up.

### Sociodemographic and health-related covariates

Self-reported sociodemographic characteristics included gender, race/ethnicity (Hispanic, Mexican American, non-Hispanic White, non-Hispanic Black, and others), marital status (married/living with a partner and separated/divorced/widowed/never married), educational level (high school or less, and more than high school), and family poverty income ratio level (< 1.30, 1.3 to < 3.5, ≥ 3.5). we also collected data on alcohol consumption, smoking status, and total Healthy Eating Index-2015 score (HEI-2015, collected through 24-h dietary recall). HEI-2015 is indicative of overall dietary quality, and the value ranges between 0–100 (worst-best). For cigarette smoking status, participants were grouped as current smokers (smoking cigarettes daily or frequently), never smokers (fewer than one hundred cigarettes throughout their entire life), or former smokers (not currently smoking but haves smoked more than 100 cigarettes throughout their entire life). Based on drinking status, participants were classified as never drinkers (had < 12 drinks throughout their life) and current drinkers (currently drinking, daily or frequently). A history of CVDs (coronary heart disease, congestive heart failure, heart attack, angina, and stroke). Diabetes was self-reported by participants who had been previously diagnosed with diabetes or if they were taking prescribed medications for diabetes (24).

### Statistical analysis

All statistical analyses were performed in R (v.4.2.2) (25) and were carried out following the analytical guidelines provided by NHANES. Survey analyses were weighted to account for sample weights to guarantee nationally representative estimates. A 2-sided p-value of < 0.05 indicated the statistically significance level.

The sociodemographic and lifestyle factors of participants were overall described by BMI categories. The differences in characteristics by BMI categories were analyzed using linear regression models. The association between BMI and all-cause, cause-specific mortality adjusted for covariates was described by means of restricted cubic spine analysis. Hazard ratios (HRs) and 95% CIs for the associations of BMI categories with all-cause, cancer, CVD and non-cancer, non-CVD mortality, were evaluated using multivariable Cox proportional hazards regression models. Cox models were adjusted for covariates including age, sex, race, family poverty income ratio, marital status, education, alcohol consumption, smoking status, HEI-2015 score, cardiovascular disease, diabetes, years since diagnosis, and cancer types. Additionally, subgroup analyses were conducted by sex, age, smoking status, and cancer type.

## Results

### Baseline characteristics

A total of 4135 cancer survivors representing 18.4 million noninstitutionalized residents of the United States were recruited in the analyzed cohort. **Table 1** includes the participant characteristics by BMI categories. The age of the participants ranged between 20–85 years. A total of 1829 (44.2%) participants reported an age > 70 years. The majority of participants were non-Hispanic White 2893(70.0%), married/living with a partner 2545(61.5%) and had more than a high school level of education 2232(54.0%). Among 4135 cancer survivors, 1486 (35.9%) were obese (21.0% class 1 obesity, 9.2% class 2 obesity, and 5.7% class 3 obesity, 1475(35.7%) were overweight, 1106 (26.7%) had normal weight, and 68 (1.6%) were underweight. Although the difference of the prevalence of obesity between female (36.4%) and male (34.7%) participants was not statistically significant, men (41.6%) had a higher prevalence of being overweight than women (29.0%). Cancer survivors aged between 55–70 years (40.6%) had a higher prevalence of obesity than those < 55 years (36.6%) and > 70 years (29.5%). A higher prevalence of obesity was observed in Mexican American (53.4%) participants than that in non-Hispanic Black participants (47.8%), Hispanic (38.1%), and non-Hispanic White participants (34.6%). Obesity was more predominant among cancer survivors with a lower family-income-to-poverty ratio as well as those with CVD and diabetes.

**Table 1:** Sample size ^a^ and characteristics of cancer survivors by BMI in the NHANES 1999 to 2018.

Characteristic	All (n=4135)	Underweight (n=68)	Normal (n=1106)	Overweight (n=1475)	Obesity 1 (n=869)	Obesity 2 (n=382)	Obesity 3 (n=235)	*P*-value^b^
Mean (SD) BMI (kg/m2)	28.7(0.1)	17.3(0.2)	22.5(0.1)	27.4(0.0)	32.2(0.1)	37.0(0.1)	44.9(0.4)	< 0.0001
Age(years), %								< 0.0001
<55	901(21.8)	16(37.3)	271(34.0)	259(26.3)	177(28.7)	104(33.1)	74(37.0)	
55-70	1405(34.0)	19(22.3)	319(32.5)	469(34.6)	326(39.3)	155(41.3)	117(47.3)	
>70	1829(44.2)	33(40.4)	516(33.5)	747(39.1)	366(32.0)	123(25.6)	44(15.8)	
Gender, %								< 0.0001
Female	2216(53.6)	49(85.1)	634(65.6)	666(48.9)	448(53.5)	245(62.4)	174(72.9)	
Male	1919(46.4)	19(14.9)	472(34.4)	809(51.1)	421(46.5)	137(37.6)	61(27.1)	
Race/ethnicity, %								< 0.0001
Hispanic	221(5.3)	3(3.4)	47(2.0)	88(2.5)	47(2.2)	25(3.8)	11(2.0)	
Mexican American	296(7.2)	2(0.6)	46(1.2)	106(2.0)	79(3.2)	40(3.4)	23(3.8)	
Non-Hispanic Black	552(13.3)	11(4.1)	106(3.3)	179(4.9)	133(6.1)	71(7.3)	52(9.2)	
Non-Hispanic White	2893(70.0)	48(89.4)	835(88.8)	1058(87.8)	578(85.3)	234(83.1)	140(81.8)	
Other	173(4.2)	4(2.4)	72(4.7)	44(2.7)	32(3.2)	12(2.3)	9(3.2)	
Marital status, %								0.02
Married or living with partner	2545(62)	35(54.2)	657(64.4)	952(70.9)	540(68.0)	233(67.4)	128(60.4)	
widowed/divorced/separated/never married	1560(38)	32(45.8)	436(35.6)	515(29.1)	323(32.0)	147(32.6)	107(39.6)	
Education, %								0.1
High school or less	1898(46.0)	36(50.8)	448(32.6)	703(38.2)	416(37.7)	181(35.2)	114(39.8)	
More than high school	2232(54.0)	32(49.2)	656(67.4)	770(61.8)	452(62.3)	201(64.8)	121(60.2)	
Mean (SD) HEI score^c^	52.9(0.3)	54.3(2.5)	54.5(0.5)	53.8(0.5)	51.3(0.5)	49.7(0.9)	50.3(1.0)	< 0.0001
Family poverty income ratio, %								< 0.0001
<1.3	899(23.8)	26(40.1)	208(13.4)	290(12.7)	200(17.0)	94(20.1)	81(27.2)	
1.3 to <3.5	1515(40.1)	20(28.5)	397(33.9)	553(37.2)	317(35.4)	141(39.4)	87(36.8)	
≥3.5	1367(36.2)	17(31.4)	405(52.7)	503(50.1)	276(47.6)	112(40.6)	54(36.0)	
Smoking, %								< 0.0001
Never smoker	1844(44.6)	17(27.3)	512(46.3)	651(45.3)	380(43.1)	176(44.7)	108(47.2)	
Former smoker	1646(39.8)	23(28.5)	366(31.7)	621(41.0)	374(41.9)	168(45.0)	94(35.9)	
Current smoker	642(15.5)	28(44.2)	227(22.0)	201(13.7)	115(15.0)	38(10.3)	33(16.9)	
Alcohol								0.002
Never drinker	509(13.4)	6(7.2)	128(9.8)	188(11.9)	105(10.0)	45(10.0)	37(14.1)	
Former drinker	956(25.1)	21(30.5)	224(17.9)	337(19.7)	207(22.9)	94(20.7)	73(31.7)	
Current drinker	2341(61.5)	33(62.3)	670(72.2)	837(68.5)	477(67.1)	216(69.3)	108(54.1)	
Cancer type, %								< 0.001
Obesity-related	1393(33.7)	25(35.1)	355(30.1)	468(27.4)	295(28.5)	141(27.9)	109(39.7)	
Non-obesity-related	2742(66.3)	43(64.9)	751(69.9)	1007(72.6)	574(71.5)	241(72.1)	126(60.3)	
Cardiovascular disease, %								0.01
No	3190(77.1)	52(77.0)	906(86.3)	1117(80.3)	635(78.0)	300(80.5)	180(79.7)	
Yes	945(22.9)	16(23.0)	200(13.7)	358(19.7)	234(22.0)	82(19.5)	55(20.3)	
Diabetes, %								< 0.0001
Yes	824(20.1)	4(4.0)	108(6.5)	265(13.9)	234(22.0)	126(30.5)	87(34.9)	
no	3285(79.9)	64(96.0)	993(93.5)	1198(86.1)	632(78.0)	252(69.5)	146(65.1)	
Years since diagnosis, %								0.3
<2	591(14.3)	12(20.1)	164(15.2)	204(13.4)	144(16.3)	41(13.2)	26(9.5)	
2 to <5	768(18.6)	11(12.1)	177(17.2)	282(19.9)	171(16.9)	77(18.9)	50(24.2)	
5 to<10	965(23.3)	9(11.0)	267(22.9)	343(22.8)	209(24.1)	82(21.3)	55(24.7)	
>=10	1811(43.8)	36(56.7)	498(44.7)	646(43.8)	345(42.7)	182(46.6)	104(41.5)	
Cause of death, %								0.003
Cancer	392(9.5)	13(16.6)	102(6.6)	154(8.0)	81(6.8)	32(6.8)	10(3.2)	
Cardiovascular disease	356(8.6)	6(8.0)	97(6.1)	146(7.4)	69(5.7)	21(3.9)	17(7.6)	
Noncancer, non-CVD	613(14.8)	16(20.1)	212(12.8)	212(10.7)	105(9.0)	46(9.2)	22(10.1)	

a weighted to be nationally representative.

b significance determined by survey-weighted one-way analysis of variance.

c Healthy Eating Index-2015 score based on the NHANES dietary data.

### BMI categories and survival

**Figure 1:**
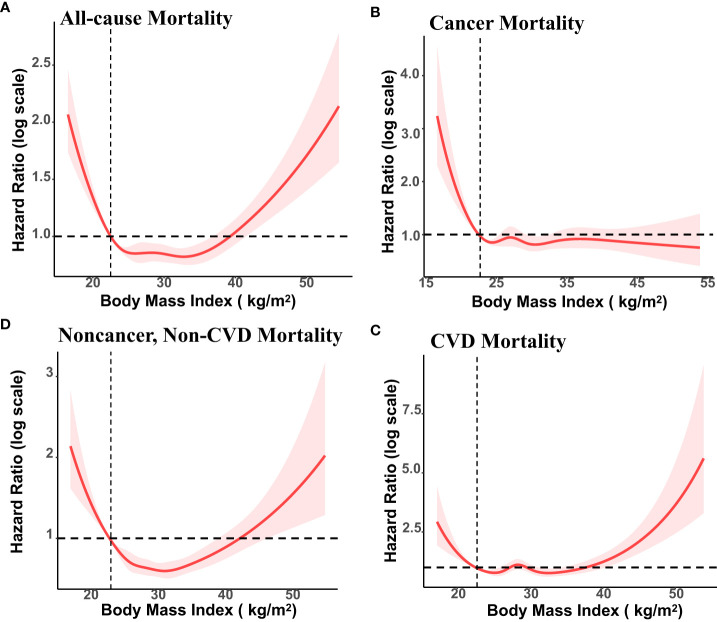
Association of Body Mass Index (BMI) with All-cause, Cancer, Cardiovascular Disease (CVD) and Non-cancer, Non-CVD Mortality Among US cancer Survivors, NHANES 1999 to 2018. BMI of 22.5 kg/m2 was the reference value. The vertical axis represents hazard ratio of mortality (log scale). Solid lines represent hazard ratios (HRs) calculated in restricted cubic spline Cox proportional hazards regression adjusted for age, ethnicity, education, family poverty income ratio, marital status, smoking status, alcohol consumption, HEI-2015 score, cardiovascular disease, and diabetes, years since diagnosis and cancer types.

During an average 8.9 years of follow-up (35895 person-years), 1361 deaths occurred; 356 patients died of CVD, 392 of cancer, and 613 of non-cancer, non-CVD causes. **Figure 1** presents the association of baseline BMI with mortality from all-cause, cancer, CVD, and non-cancer, non-CVD. The relationship of BMI with mortality was U-shaped for all-cause and non-cancer, non-CVD mortality. While there was no significant correlation between whole obesity group and mortality, class 1 obesity was related to significantly decreased mortality as compared to normal weight from all-cause mortality (HR, 0.78; 95% CI, 0.61-0.99) among cancer survivors after adjusting for covariates (**Table 2**).

**Table 2:** Body mass index (BMI) and cause-specific mortality in the multiethnic cohort, NHANES 1999 to 2018.

Mortality outcome	Death, n	Hazard Ratio (95% CI)		
		Model 1^a^	Model 2^a,b^	Model 3^a,b,c^
All-cause				
Underweight	35	2.19(1.34,3.61) **	1.43(0.73,2.81)	1.40(0.70,2.80)
Normal	411	1.00(reference)	1.00(reference)	1.00(reference)
Overweight	512	0.88(0.75,1.02)	0.86(0.72,1.02)	0.86(0.72,1.03)
Obese	403	0.97(0.80,1.16)	0.86(0.69,1.07)	0.87(0.70,1.08)
Obese class 1	255	0.86(0.70,1.06)	0.77(0.61,0.98) *	0.78(0.61,0.99) *
Obese class 2	99	1.05(0.80,1.37)	0.94(0.69,1.29)	0.95(0.69,1.31)
Obese class 3	49	1.42(0.98,2.05)	1.19(0.79,1.80)	1.20(0.80,1.80)
P for trend		0.96	0.41	0.46
Cancer				
Underweight	13	4.10(1.86,9.05) **	3.15(1.35,7.33) *	3.31(1.37,8.03) *
Normal	102	1.00(reference)	1.00(reference)	1.00(reference)
Overweight	154	0.97(0.71,1.31)	0.87(0.61,1.25)	0.88(0.61,1.26)
Obese	123	0.94(0.67,1.32)	0.83(0.55,1.27)	0.83(0.54,1.28)
Obese class 1	81	0.89(0.60,1.33)	0.81(0.50,1.33)	0.82(0.50,1.34)
Obese class 2	32	1.22(0.76,1.96)	1.08(0.64,1.82)	1.06(0.62,1.82)
Obese class 3	10	0.65(0.31,1.37)	0.56(0.25,1.25)	0.55(0.25,1.25)
P for trend		0.48	0.25	0.25
CVD				
Underweight	6	4.01(1.71,9.39) **	3.32(1.50,7.38) **	3.18(1.44,7.02) **
Normal	97	1.00(reference)	1.00(reference)	1.00(reference)
Overweight	146	0.97(0.71,1.32)	0.98(0.69,1.39)	1.00(0.70,1.43)
Obese	107	1.14(0.80,1.62)	0.91(0.60,1.39)	0.95(0.63,1.45)
Obese class 1	69	0.97(0.67,1.40)	0.78(0.51,1.21)	0.81(0.52,1.26)
Obese class 2	21	1.01(0.57,1.77)	0.76(0.40,1.43)	0.80(0.42,1.52)
Obese class 3	17	2.89(1.41,5.90) **	2.21(1.00,4.89) *	2.35(1.07,5.18) *
P for trend		0.26	0.85	0.68
Noncancer, non-CVD disease				
Underweight	16	1.92(1.12,3.29) *	1.24(0.58,2.66)	1.21(0.53,2.74)
Normal	212	1.00(reference)	1.00(reference)	1.00(reference)
Overweight	212	0.70(0.56,0.87) **	0.66(0.50,0.86) **	0.66(0.51,0.87) **
Obese	173	0.84(0.65,1.07)	0.70(0.52,0.94) *	0.71(0.52,0.96) *
Obese class 1	105	0.72(0.53,0.97) *	0.60(0.42,0.85) **	0.60(0.42,0.86) *
Obese class 2	46	0.93(0.65,1.35)	0.80(0.50,1.26)	0.81(0.51,1.29)
Obese class 3	22	1.36(0.82,2.26)	1.02(0.59,1.78)	1.03(0.59,1.77)
P for trend		0.31	0.06	0.08

**P < 0.01; *P < 0.05, Significant results (*P* < 0.05) are indicated in bold.

a Adjusted for age, sex and ethnicity.

b Multivariable model additionally adjusted for education, family poverty income ratio, marital status, smoking status, alcohol consumption, HEI-2015 score, cardiovascular disease, and diabetes.

^C^Additionally adjusted for years since diagnosis and cancer types.

For CVD mortality, both underweight (HR, 3,18;95% CI, 1.44-7.02) and class 3 obesity (HR, 2.35; 95% CI, 1.07-5.18) showed a significant positive association with higher rates of mortality. For cancer mortality, underweight group was associated with an elevated risk of mortality (HR, 3.31; 95% CI, 1.37-8.03). The association between cancer mortality with overweight and obesity did not reach significantly difference. For non-cancer, non-CVD mortality, overweight and class 1 obesity were found to be associated with a significantly lower number of excess deaths, with HRs of 0.66(0.51,0.87) and 0.60 (0.42,0.86), respectively.

### Subgroup analyses

The stratified analysis was conducted by age at in-person interview (<55, 55–70, and >70 years), cancer types of survivors (obesity-related and non-obesity-related cancers), and smoking status (former smokers, never-smokers, and current smokers). We observed an association of overweight and class 1 obesity with reduced mortality among male survivors, but the association was not significant in women (**Table 3**). Compared to the normal weight group, cancer survivors (age older than 70 years) who were overweight and class 1 obese had better overall survival. Cancer survivors with class 2 obesity (age < 55 years) and cancer survivors (at the age of 55-70 years) with class 1 obesity had a reduced risk in all-cause mortality (**Table 4**). Overweight among former smokers or class 1 obesity among never-smokers had a lower risk of all-cause mortality, while overweight or obese current smokers did not have a significantly reduced risk of mortality. However, being underweight was associated with an elevated risk for all-cause mortality in current smokers (**Table 5**). Among survivors with diagnoses of obesity-related cancers, individuals with class 2 obesity showed a decreased risk of all-cause mortality (HR, 0.49; 95% CI, 0.27-0.87). For survivors with diagnoses of non-obesity-related cancers, being underweight was correlated with a higher risk of all-cause mortality (**Table 6**). A further exploration of the association of BMI with the death risk for each specific cancer and mortality was conducted (**eTable 1** in the Supplement). For prostate cancer survivor, being overweight was associated with reduced risk in all-cause mortality (HR, 0.57; 95% CI, 0.39-0.82).

**Table 3:** Body mass index (BMI) and total mortality in the multiethnic cohort, by sex, NHANES 1999 to 2018^a^.

BMI	Male(n=956)		Female(n=698)	
	Death, n	HR (95%CI)	Death, n	HR (95%CI)
Underweight	11	2.36(0.91,6.12)	24	1.32(0.63,2.79)
Normal	220	1.00(reference)	191	1.00(reference)
Overweight	333	0.76(0.59,0.99) *	179	0.94(0.75,1.18)
Obese	210	0.81(0.58,1.13)	193	0.91(0.70,1.18)
Obese class 1	149	0.69(0.49,0.98) *	106	0.88(0.63,1.24)
Obese class 2	47	1.05(0.66,1.66)	52	0.89(0.62,1.28)
Obese class 3	14	1.75(0.80,3.86)	35	1.04(0.69,1.56)
P for trend		0.565		0.519

HR, hazard ratio; *P < 0.05. Significant results (*P* < 0.05) are indicated in bold.

a Multivariable model adjusted for age, ethnicity, education, family poverty income ratio, marital status, smoking status, alcohol consumption, HEI-2015 score, cardiovascular disease, and diabetes, years since diagnosis and cancer types.

**Table 4:** Body mass index (BMI) and total mortality in the multiethnic cohort, by age group, NHANES 1999 to 2018^a^.

BMI	<55y(n=66)		55-70y(n=330)		>70y(n=965)	
	Death, n	HR (95%CI)	Death, n	HR (95%CI)	Death, n	HR (95%CI)
Underweight	2	2.96(0.47,18.58)	8	1.81(0.70,4.65)	25	1.30(0.59,2.83)
Normal	21	1.00(reference)	87	1.00(reference)	303	1.00(reference)
Overweight	18	0.66(0.25, 1.76)	104	0.85(0.55,1.31)	390	0.87(0.71,1.05)
Obese	25	1.19(0.53, 2.67)	131	0.76(0.48,1.19)	247	0.74(0.57,0.95) *
**Obese class 1**	**11**	**0.88(0.30, 2.62)**	**69**	0.61(0.39,0.96) *	**175**	0.74(0.55,0.98) *
Obese class 2	11	2.70(1.07, 6.83) *	33	0.89(0.48,1.66)	55	0.68(0.47,0.98) *
Obese class 3	3	0.50(0.14, 1.80)	29	1.14(0.59,2.23)	17	0.92(0.53,1.60)
P for trend		0.724		0.650		0.02

HR, hazard ratio, *P < 0.05. Significant results (*P* < 0.05) are indicated in bold.

a Multivariable model adjusted for sex, ethnicity, education, family poverty income ratio, marital status, smoking status, alcohol consumption, HEI-2015 score, cardiovascular disease, and diabetes, years since diagnosis and cancer types.

**Table 5:** Body mass index (BMI) and total mortality in the multiethnic cohort, by smoking status, NHANES 1999 to 2018^a^.

BMI	Never-Smokers(n=519)		Former-Smokers (n=664)		Current-Smokers (n=178)	
	Death, n	HR (95%CI)	Death, n	HR (95%CI)	Death, n	HR (95%CI)
Underweight	5	1.59(0.85,2.97)	15	1.13(0.41,3.08)	15	2.84(1.24,6.52) *
Normal	171	1.00(reference)	172	1.00(reference)	68	1.00(reference)
Overweight	202	0.92(0.72,1.18)	258	0.77(0.60,0.98) *	52	0.87(0.51,1.46)
Obese	141	**0.68(0.48,0.97)**	219	0.88(0.66,1.17)	43	1.11(0.63,1.95)
Obese class 1	92	0.61(0.41,0.90) *	132	0.79(0.58,1.09)	31	1.00(0.54,1.87)
Obese class 2	31	0.79(0.48,1.30)	61	0.94(0.61,1.46)	7	1.02(0.25,4.23)
Obese class 3	18	0.86(0.44,1.69)	26	1.23(0.75,2.04)	5	1.50(0.43,5.27)
P for trend		0.089		0.659		0.840

HR, hazard ratio; *P < 0.05. Significant results (*P* < 0.05) are indicated in bold.

a Multivariable model adjusted for age, sex, ethnicity, education, family poverty income ratio, marital status, alcohol consumption, HEI-2015 score, cardiovascular disease, and diabetes, years since diagnosis and cancer types.

**Table 6:** Body mass index (BMI) and total mortality in the multiethnic cohort, by cancer types, NHANES 1999 to 2018^a^.

BMI	Obesity-related cancer(n=469)		Non−obesity-related cancer(n=892)	
	Death, n	HR (95%CI)	Death, n	HR (95%CI)
Underweight	11	0.70(0.33,1.47)	24	**3.39(1.88,6.10)** **
Normal	220	1.00(reference)	191	1.00(reference)
Overweight	333	0.80(0.59,1.10)	179	0.86(0.68,1.08)
Obese	210	0.74(0.53,1.03)	193	0.94(0.73,1.22)
Obese class 1	149	0.75(0.51,1.10)	106	0.79(0.60,1.04)
Obese class 2	47	0.49(0.27,0.87) *	52	1.25(0.85,1.86)
Obese class 3	14	1.07(0.64,1.78)	35	1.25(0.69,2.27)
P for trend		0.164		0.917

HR, hazard ratio; **P < 0.01*P < 0.05. Significant results (*P* < 0.05) are indicated in bold.

a Multivariable model adjusted for age, sex, ethnicity, education, family poverty income ratio, marital status, smoking status, alcohol consumption, HEI-2015 score, cardiovascular disease, and diabetes, and years since diagnosis.

## Discussion

In this cohort of cancer survivors from the United States, nearly three-quarters of the participants were identified as overweight or obese. This study evaluated the association of BMI categories with survival outcomes among cancer survivors. Over an 8.9-years of follow-up period, underweight status was significantly associated with increased mortality risk in both cancer and CVD cases, but not in all-cause, or non-cancer, non-CVD mortality. Overweight and moderate obesity were linked with a reduced risk of all-cause and non-cancer, non-CVD mortality in cancer survivors after accounting for confounding factors such as smoking, co-morbid conditions, and other covariates. This finding suggests the existence of an “obesity paradox”. Comparable associations were observed among the never-smoker and former smoker group, but not in current smokers; in male participants, but not in female participants; in obesity-related cancers, but not in non-obesity-related cancers. Class 3 obesity was associated with elevated risks for CVD mortality.

Among cancer survivors, an increased BMI has been association with survival benefits compared with normal-weight patients. The obesity paradox was observed in various types of cancer, including in patients undergoing surgery for stages I-III colorectal cancer (12), patients undergoing renal mass surgery (14), patients who had liver resection for colorectal cancer metastases (26); elderly patients receiving chemotherapy for acute myeloid leukemia (27); and cancer patients with distant metastases who received radiotherapy (28). In alignment with previous studies, our research demonstrated a reduced risk of all-cause and non-cancer, non-CVD death among cancer survivors with overweight or class 1 obesity. Interestingly, the association seem to vary based on gender; consistent with this study, the reverse association was more prevalent in men than in women (29). Such differences may arise from variations in disease mechanisms at the cellular and molecular level or differing reactions to treatment. The disparity in the muscle-to-fat ratio may help explain the gender differences (30, 31). Further investigation is required to explore these differences. Nevertheless, some researchers have argued that inverse associations may result from methodological limitations including reverse causality, collider bias, or confounding by smoking and comorbidities (16, 32, 33).

Reverse causality might arise when weight loss results from cancer rather than being its cause. To minimize the possibility of reverse causality in this study, patients who died within the initial 24 months of follow-up were excluded. With adequate covariate adjustment, including smoking and comorbidities, the prognosis was found to be most favorable for cancer survivors who were overweight or had class 1 obesity. Moreover, survivors with class 2 obesity and obesity-related cancer exhibited better overall prognosis compared to normal-weight patients, although similar statistically significant associations were not observed among non-obesity-related cancer survivors. A strength of the present study is the comprehensive adjustment for smoking, a potent cancer risk factor that could lead to an inverse association with mortality. When examining the association between BMI and all-cause mortality stratified by smoking status, the obesity paradox persisted among never smokers and former smokers. The associations between BMI and the leading causes of death were also investigated. Reduced mortality among overweight and class 1 obese patients was primarily associated with non-cancer, non-CVD causes, rather than cancer or CVD. It appears that confounding by smoking and comorbidities or collider bias may not explain the survival benefits observed among cancer survivors with overweight and moderately obese BMI.

Limited sample sizes and broad weight group categorizations may have led to the absence of associations between obesity and outcomes in previous studies (7, 34). Some studies have redefined BMI categories, such as obese class 1 (BMI 25-29.9kg/m^2^), obese class 2 (BMI ≥ 30kg/m^2^) (35), while others have used standard WHO criteria (obese BMI > 30.0kg/m^2^), which is a broad categorization. In the current study, obesity was divided into three categories. Class 1 obesity, rather than whole obesity or class 2/3 obesity, was significantly associated with reduced mortality risks of all-cause and non-cancer, non-CVD causes, suggesting that existing standard BMI categories are insufficiently refined to accurately assess mortality risk in similar studies.

Several potential explanations for the obesity paradox appear biologically plausible. Within the context of disease, survival benefits of being overweight or obese could be attributed to improved nutritional status (36), reduced thromboxane B2 levels (37), and enhanced mobilization of endothelial progenitor cells (38). Additionally, certain tumor subtypes might be less aggressive in overweight or obese cancer survivors. For instance, clear-cell renal cell carcinoma in obese patients may be more indolent compared to normal-weight patients due to differential expressions of metabolic and fatty acid genes (14). Moreover, overweight and obese cancer survivors may respond differently to treatment and potentially benefiting from the influence on treatment outcomes. Among these cancer survivors, excess adipose tissue serves as nutrient reserves, helping to counteract decreased energy intake and increased demands during cancer progression and treatment (39).

## Strengths and limitations

One of the primary strengths of the study is the use of NHANES, which allowed for access to a large and nationally representative sample. Additionally, a wide range of confounding factors, including age, race/ethnicity, sex, education, family poverty income ratio, HEI, marital status, alcohol intake, smoking consumption, diabetes, CVD, and cancer type, were adjusted in the analyses. However, there are certain limitations that must be considered. Covariates were assessed at baseline, and these may have changed significantly during the follow-up period. A one-time BMI measure, not taken before or near the time of cancer diagnosis, does not reflect the cumulative impact of being overweight or obese on cancer survival. Moreover, there was insufficient data on cancer stage, histology type, and treatments. Nevertheless, the obesity paradox was still observed after excluding deaths in the initial 24-month follow-up period.

## Conclusions

In this prospective cohort study of cancer survivors in the United States, it was discovered that being underweight or extremely obese was associated with a heightened risk for mortality, primarily in cancer and CVD-related causes. Overweight or mildly obese conditions were associated with significantly reduced mortality from all-cause and non-cancer, non-CVD causes and were not associated with mortality from cancer and CVD. Therefore, this study demonstrates that the associated of BMI with mortality varies substantially depending on the cause of death. To provide comprehensive survivorship care, future efforts are needed to investigate the effect of body weight on different outcomes among cancer survivors.

## Data availability statement

The datasets presented in this study can be found in online repositories. The names of the repository/repositories and accession number(s) can be found in the article/**Supplementary Material**.

## Ethics statement

Ethical review and approval was not required for the study on human participants in accordance with the local legislation and institutional requirements. The patients/participants provided their written informed consent to participate in this study.

## Author contributions

YY designed the study, analyzed and wrote the manuscript. DC and DZ provided the statistical analyses. YZ provided critical revision of the manuscript. All authors contributed to the article and approved the submitted version.
