# Supplementary material for: Association of body mass index with survival in U.S. cancer survivors: a cross-sectional study of NHANES 1999–2018
Source: Front Oncol. 2023 May 12;13:1180442. doi: 10.3389/fonc.2023.1180442 (PMC10213672; doi:10.3389/fonc.2023.1180442)
Supplement: Supplementary file 1 [file DataSheet_1.pdf]

# Supplementary

**eTable 1.** Body Mass Index (BMI) and Total Mortality in the Multiethnic Cohort, by specific cancer, NHANES 1999 to 2018[a]

| BMI | **Breast (n=193)** | | **Prostate (n=260)** | | **Skin (non-melanoma) (n=185)** | |
|---|---|---|---|---|---|---|
| | **Death, n** | HR (95%CI) | **Death, n** | HR (95%CI) | **Death, n** | HR (95%CI) |
| Underweight | 6 | 0.69(0.28,1.73) | 4 | 0.89(0.31,2.61) | 4 | 1.35(0.33, 5.54) |
| Normal | 77 | 1.00(reference) | 73 | 1.00(reference) | 58 | 1.00(reference) |
| Overweight | 55 | 0.69(0.45,1.06) | 105 | **0.57(0.39,0.82)** ** | 73 | 0.93(0.60, 1.44) |
| Obese class 1 | 40 | 0.82(0.48,1.41) | 58 | 0.86(0.50,1.49) | 37 | 1.11(0.64, 1.94) |
| Obese class 2 | 13 | 0.42(0.17,1.05) | 17 | 1.88(0.85,4.13) | 10 | 1.16(0.59, 2.31) |
| Obese class 3 | 12 | 1.84(0.94,3.59) | 3 | 0.93(0.18,4.80) | 3 | 1.05(0.17, 6.61) |
| | Uterus **(n=47)** | | Lung **(n=41)** | | Cervix **(n=31)** | |
| | **Death, n** | HR (95%CI) | **Death, n** | HR (95%CI) | **Death, n** | HR (95%CI) |
| Underweight | 1 | 1.71(0.15, 19.49) | 2 | 0.27(0.01, 11.03) | 2 | 1.04(0.05, 22.54) |
| Normal | 8 | 1.00(reference) | 11 | 1.00(reference) | 13 | 1.00(reference) |
| Overweight | 16 | 1.48(0.27, 8.20) | 15 | 0.92(0.28, 3.05) | 4 | 0.36(0.06, 2.12) |
| Obese class 1 | 10 | 0.23(0.02, 2.53) | 9 | 0.45(0.10, 2.16) | 4 | 0.07(0.00, 0.97) |
| Obese class 2 | 3 | 0.20(0.02, 2.27) | 3 | 2.00(0.49, 8.24) | 7 | 1.11(0.30, 4.18) |
| Obese class 3 | 9 | 0.48(0.07, 3.28) | 1 | 0.30(0.01, 7.70) | 1 | 0.92(0.13, 6.37) |

HR=hazard ratio; **$P < 0.01$

[a] Multivariable model adjusted for age, ethnicity, education, family poverty income ratio, marital status, smoking status, alcohol consumption, HEI-2015 score, cardiovascular disease, and diabetes, years since diagnosis and cancer types.
